# The association between culture positivity and long-term mortality in critically ill surgical patients

**DOI:** 10.1186/s40560-021-00576-2

**Published:** 2021-10-26

**Authors:** Yu-Cheng Wu, Li-Ting Wong, Chieh-Liang Wu, Wen-Cheng Chao

**Affiliations:** 1 Division of Chest Medicine, Department of Internal Medicine, Taichung Veterans General Hospital, Taichung, Taiwan; 2 Department of Medical Research, Taichung Veterans General Hospital, Taichung, Taiwan; 3 Department of Critical Care Medicine, Taichung Veterans General Hospital, No, 1650, Section 4, Taiwan Boulevard, Xitun District, 40705 Taichung, Taiwan; 4 Department of Computer Science, Tunghai University, Taichung, Taiwan; 5 Department of Automatic Control Engineering, Feng Chia University, Taichung, Taiwan; 6 Department of Industrial Engineering and Enterprise Information, Tunghai University, Taichung, Taiwan; 7 Artificial Intelligence Studio, Taichung Veterans General Hospital, Taichung, Taiwan

**Keywords:** Critical illness, Long-term outcome, Surgery, Survival analysis, Culture positivity

## Abstract

**Background:**

The long-term outcome is an essential issue in critically ill patients, and the identification of early determinant is needed for risk stratification of the long-term outcome. In the present study, we investigate the association between culture positivity during admission and long-term outcome in critically ill surgical patients.

**Methods:**

We linked the 2015–2019 critical care database at Taichung Veterans General Hospital with the nationwide death registration files in Taiwan. We described the long-term mortality and proportion of culture positivity among enrolled subjects. We used a log-rank test to estimate survival curves between patients with and without positive cultures and a multivariable Cox proportional hazards regression model to determine hazard ratio (HR) and 95% confidence interval (CI).

**Results:**

A total of 6748 critically ill patients were enrolled, and 32.5% (2196/6749) of them died during the follow-up period, with the overall follow-up duration was 1.8 ± 1.4 years. We found that 31.4% (2122/6748) of critically ill patients had at least one positive culture during the index admission, and the number of patients with positive culture in the blood, respiratory tract, urinary tract, skin and soft tissue and abdomen were 417, 1702, 554, 194 and 139, respectively. We found that a positive culture from any sites was independently associated with high long-term mortality (aHR 1.579, 95% CI 1.422–1.754) after adjusting relevant covariates, including age, sex, body-mass index, comorbidities, severity score, shock, early fluid overload, receiving mechanical ventilation and the need of renal replacement therapy for critical illness.

**Conclusions:**

We linked two databases to identify that a positive culture during admission was independently correlated with increased long-term mortality in critically ill surgical patients. Our findings highlight the need for vigilance among patients with a positive culture during admission, and more studies are warranted to validate our findings and to clarify underlying mechanisms.

**Supplementary Information:**

The online version contains supplementary material available at 10.1186/s40560-021-00576-2.

## Background

The long-term outcome is an emerging issue in critically ill patients; however, evidence focusing on critically ill surgical patients remains sparse [1–3]. A number of studies have explored that the 1-year mortality among patients discharged from intensive care units (ICUs) was approximately 15–35%, and the data varied due to high heterogeneity among ICUs [4–6]. Studies are required to address long-term outcomes and identify early determinants for risk stratification in critically ill surgical patients. Increasing evidence have shown the crucial role of chronic critical illness (CCI), characterised by persistent organ dysfunction, vulnerability for secondary infection, prolonged ICU course and high resource utilisation after discharge, among critically ill surgical patients [7]. A number of studies have demonstrated the critical illness-associated prolonged microbial alternation among critically ill surgical patients [8–10]. One recent study further characterised CCI with the prolonged immunological and metabolic alternation in 144 critically ill surgical patients with abdominal infection [11]. These data highlight the need to identify early determinants, particularly microbial factors, of the long-term outcome in surgical patients requiring intensive care.

Recent studies have explored the association between culture positivity, a positive microbial culture of clinical samples during admission, and mortality in critically ill patients, but the evidence appeared to be inconclusive due to high heterogeneity among studies, with the majority of studies explored the short-term impact of culture positivity among patients with sepsis in the medical ICU [12–14]. Few studies, including our previous study focusing on 638 patients with cancer receiving perioperative intensive care, have found that culture positivity tended to be associated with 1-year mortality instead of short-term mortality [15, 16].

In the present study, we linked the critical care database at Taichung Veteran General Hospital (TCVGH) and the death registration data of the Taiwanese National Health Insurance Research Database (NHIRD) to investigate the overall long-term mortality, to address main pathogens in distinct culture sites, and to identify early determinants for risk stratification of long-term mortality in critically ill surgical patients.

## Materials and methods

### Ethical approval

The present study was approved by The Institutional Review Board of the Taichung Veterans General Hospital (TCVGH: SE20249B#1) with the exemption of informed consent due to the analysed data were de-identified.

### Study population

This retrospective cohort study was conducted at TCVGH, a referral hospital with 1530 beds in central Taiwan. We enrolled consecutive patients admitted to surgical ICUs at TCVGH between 2015 and 2019. There are no strict criteria for ICU admission; however, in general, critically ill patients who need surgical intensive care, such as the patients after neurosurgery, cardiovascular surgery, major abdomen surgery, or other emergency surgery, admit to ICU. We used the first ICU admission as the index ICU admission among those with more than one ICU admission.

### Data source

We used the critical care database at TCVGH for variables, including demographic data, divisions of ICU admission, comorbidities using International Classification of Diseases, 9th and 10th Revision, Clinical Modification (ICD-9/10-CM) codes, Acute Physiology and Chronic Health Evaluation (APACHE) II score, medications including vasopressors as well as antimicrobial agents, and managements including use of mechanical ventilation and renal replacement therapy. The primary outcome of interest in the present study was the all-cause mortality following ICU admission. To ascertain the date-of-death of enrolled critically surgical patients, we linked the critical care database of TCVGH with the death registration profile of the Taiwanese National Health Insurance Research Database (NHIRD) [17]. In brief, Taiwanese National Health Insurance (NHI) is a single-payer and compulsory health insurance program with up to 99.9% coverage of the Taiwanese population in 2019.

### Microbiology cultures

The exposure of this study was the positive culture of clinical samples during the index admission. The culture cites compromised of blood, respiratory tract (sputum, tracheal aspirate, pleural effusion, and bronchoalveolar lavage fluid), urinary tract (midstream urine, urine via urinary catheter, and urine via percutaneous nephrostomy), skin and soft tissue (surgical wound, pus, and discharge), or abdomen (ascites, bile, and drainage of abdominal drainage tube) during the index admission [15]. The pathogens were categorised by Gram-negative bacilli, Gram-positive cocci, and Fungi, including *Candida* and *Aspergillus* [15]. The microbiologic test was performed based on the decision made by individual physicians if patients had a fever or an infection was suspected.

### Statistical analyses

Descriptive results were presented as means ± standard deviation or number (percentages). Kaplan–Meier analysis was performed to illustrate the association between mortality and microbial results. Variables that might affect long-term mortality in critically ill patients based on previous studies including our study were included in the multivariable model [15, 18]. The Cox proportional hazards model was used to estimate hazard ratios (HRs) and 95% confidence intervals (CIs) for mortality after adjustment for age, sex, CCI, and potential cofounders including early fluid balance, a predictor for long-term outcome in critically ill cancer patients as we have shown in our previous study [19]. Statistical analyses were two-sided, and the level of significance was set at 0.05. Data cleaning and analysis were performed using R version 3.6.0.

### Sensitivity analysis

We further defined culture positivity by positive culture for distinct numbers of pathogens and cites to test the robustness of association between culture positivity and long-term mortality in critically ill surgical patients.

### Subgroup analysis

In addition, we used the Wald test to determine the significance of modification effect by covariates, including age, gender, diabetes mellitus, presence of malignancy, and types of surgery.

## Results

### Demographic and characteristic data of the enrolled critically ill surgical patients

**Fig. 1:**
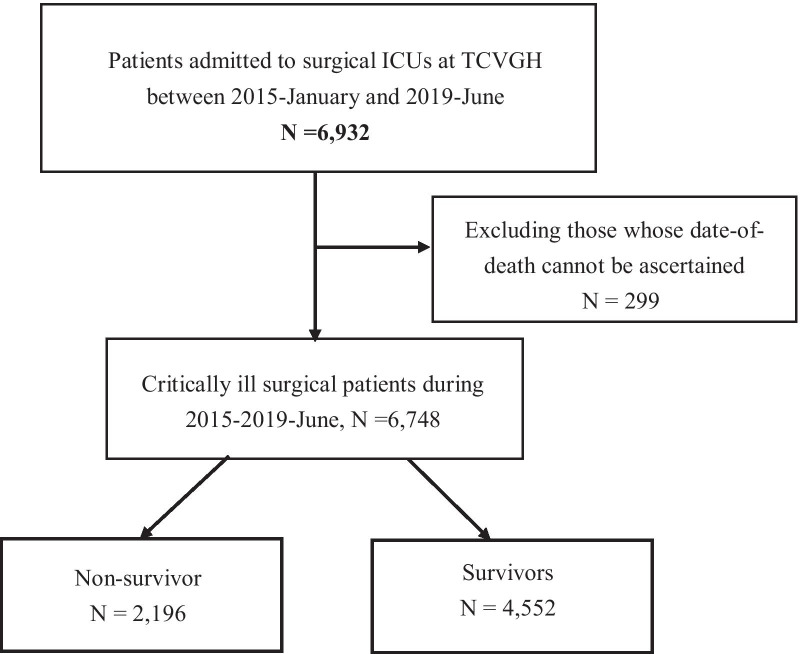
Flowchart of subject enrolment. *TCVGH* Taichung Veterans General Hospital, *ICU* intensive care unit

**Fig. 2:**
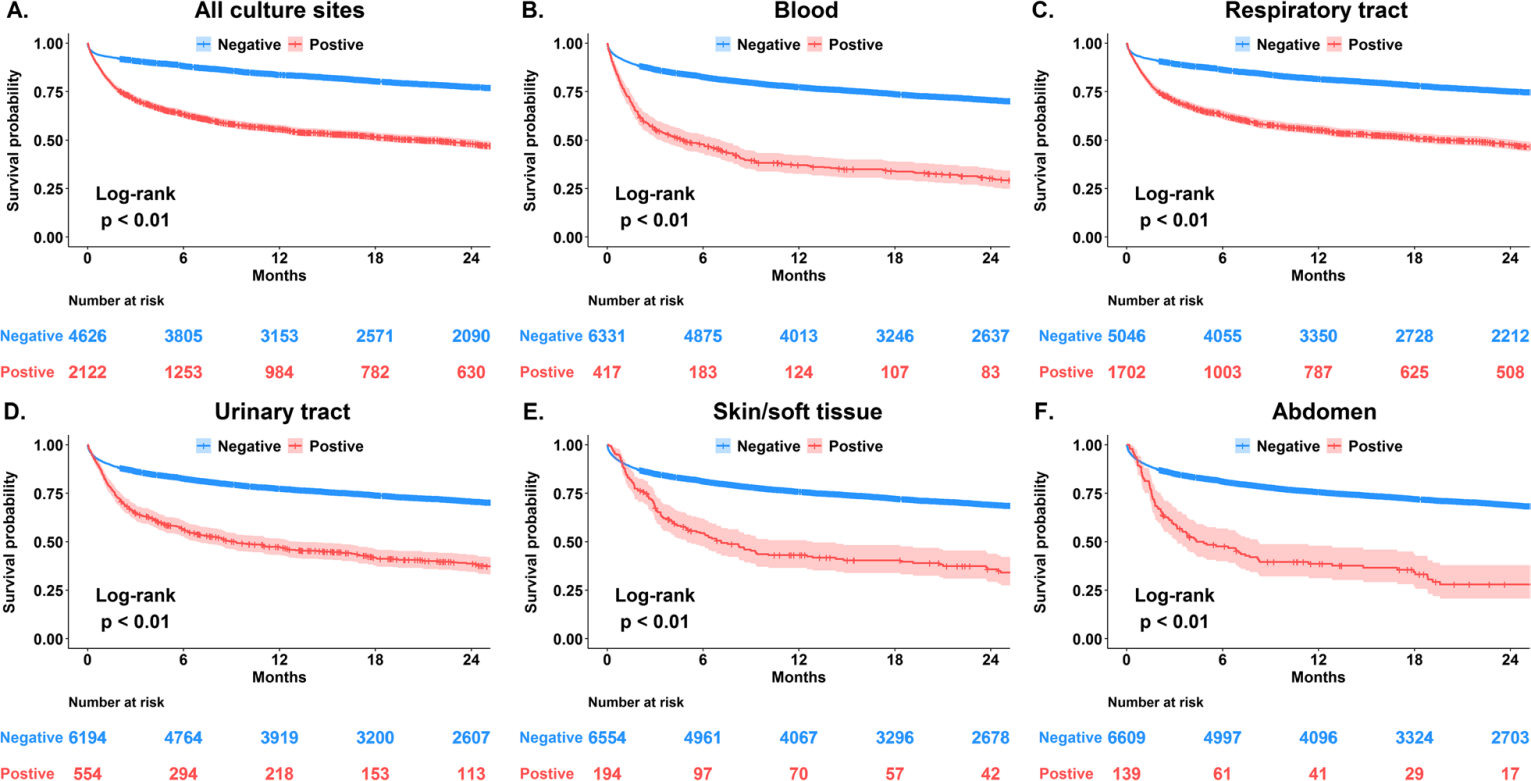
Kaplan–Meier survival curves for critically ill surgical patients with and without positive cultures categorised by culture sites. **A** All culture sites, **B** blood, **C** respiratory tract, **D** urinary tract, **E** skin or soft tissue, **F** abdomen

We enrolled 6748 patients who had been admitted to surgical ICUs at TCVGH during 2015–2019, and 64.2% of them were male, with the mean age was 60.9 ± 15.9 years (Figs. 1, 2 and Table 1). The overall follow-up duration was 1.8 ± 1.4 years, and 32.5% (2196/6749) of them died during the follow-up period. The main divisions of ICU admission compromised of neurosurgery (49.3%), followed by cardiovascular surgery (20.4%), chest surgery (7.0%), general surgery (6.8%), and colon–rectal surgery (5.6%). In the present study, the in-hospital mortality rate, 90-day and 1-year mortality was 10.4% (705/6748), 16.1% (1088/6748) and 24.4% (1648/6748), respectively. Therefore, the post-discharge 1-year mortality rate in critically ill patients who survived after the ICU admission was 15.6% (943/6043). Compared with survivors, non-survivors were older (66.4 ± 15.3 vs. 58.3 ± 15.5 years, *p* < 0.01), were more likely to be male (69.2 vs. 61.8%, *p* < 0.01), and had more comorbidities. Non-survivors had a higher APACHE II score (23.2 ± 6.6 vs. 19.0 ± 5.9, *p* < 0.01) and were more likely to have shock, which was defined by the use of vasopressor (40.6 vs. 23.8%, *p* < 0.01), to receive mechanical ventilation for more than 3 days (48.9 vs. 26.0%, *p* < 0.01) and to receive renal replacement therapy (11.5 vs. 1.9%, *p* < 0.01) (Table 1). Taken together, these data demonstrated a high post-acute mortality rate after ICU discharge in those with high disease severity and the crucial need to explore the early determinants for long-term mortality in critically ill surgical patients.

**Table 1:** Characteristics of enrolled critically ill surgical patients categorised by overall mortality

	All	Non-survivors	Survivors	*p* value
	(*N* = 6748)	(*N* = 2196)	(*N* = 4552)	
Basic characteristics				
Age, years	60.9 ± 15.9	66.4 ± 15.3	58.3 ± 15.5	< 0.01
Sex (male)	4334 (64.2%)	1519 (69.2%)	2815 (61.8%)	< 0.01
Body mass index	24.6 ± 4.5	23.8 ± 4.5	25.1 ± 4.5	< 0.01
Charlson comorbidity index	1.6 ± 1.4	2.3 ± 1.4	1.3 ± 1.2	< 0.01
Follow-up duration, years	1.8 ± 1.4	0.7 ± 0.9	2.3 ± 1.3	< 0.01
Comorbidities				
Diabetes mellitus	1580 (23.4%)	603 (27.5%)	977 (21.5%)	< 0.01
Congestive heart failure	513 (7.6%)	202 (9.2%)	311(6.8%)	< 0.01
Chronic pulmonary disease	182 (2.7%)	96 (4.4%)	86 (1.9%)	< 0.01
Moderate or severe liver disease	184 (2.7%)	98 (4.46%)	86 (1.9%)	< 0.01
End-stage renal disease	63 (1.0%)	32 (1.5%)	31 (0.7%)	< 0.01
Malignancy	1274 (18.9%)	718 (32.7%)	556 (12.2%)	< 0.01
Presence of metastatic tumour	237 (3.5%)	181 (8.2%)	56 (1.2%)	< 0.01
Divisions				< 0.01
Neurosurgery	3328 (49.3%)	839 (38.2%)	2489 (54.7%)	
Cardiovascular surgery	1376 (20.4%)	262 (11.9%)	1114 (24.5%)	
Major abdomen surgery	840 (12.4%)	465 (21.2%)	375 (8.2%)	
Chest surgery	472 (7.0%)	235 (10.7%)	237 (5.2%)	
Urological surgery	165 (2.4%)	107 (4.9%)	58 (1.3%)	
Otorhinolaryngology	200 (3.0%)	130 (5.9%)	70 (1.5%)	
Plastic surgery	95 (1.4%)	45 (2.1%)	50 (1.1%)	
Other divisions	272 (4.0%)	113 (5.2%)	159 (3.5%)	
Severity and managements				
APACHE II score	20.4 ± 6.4	23.2 ± 6.6	19.0 ± 5.9	< 0.01
Presence of shock	1975 (29.3%)	892 (40.6%)	1083 (23.8%)	< 0.01
Receiving surgery during admission	4818 (71.4%)	1364 (62.1%)	3454 (75.9%)	< 0.01
Emergent surgery	994 (14.7%)	332 (15.1%)	662 (14.5%)	0.53
Receiving mechanical ventilation	2256 (33.4%)	1074 (48.9%)	1182 (26.0%)	< 0.01
Renal replacement therapy	340 (5.0%)	253 (11.5%)	87 (1.9%)	< 0.01
Fluid balance, days 1–3, mL	1142.0 ± 2672.7	1601.4 ± 3312.8	920.3 ± 2268.5	< 0.01
Microbiologic data				
Presence of positive cultures	2122 (31.4%)	1110 (50.6%)	1012 (22.2%)	< 0.01
Culture sites				
Blood	417 (6.2%)	283 (12.9%)	134 (2.9%)	< 0.01
Respiratory tract	1702 (25.2%)	900 (41.0%)	802 (17.6%)	< 0.01
Urinary tract	554 (8.2%)	341 (15.5%)	213 (4.7%)	< 0.01
Skin and soft tissue site	194 (2.9%)	123 (5.6%)	71 (1.6%)	< 0.01
Abdomen	139 (2.1%)	95 (4.3%)	44 (1.0%)	< 0.01
Other sites	349 (5.2%)	210 (9.6%)	139 (3.1%)	< 0.01
Outcomes				
ICU-stay, days	8.7 ± 11.4	12.2 ± 15.4	7.1 ± 8.4	< 0.01
Hospital-stay, days	22.4 ± 24.3	29.5 ± 33.2	19.0 ± 17.6	< 0.01
Ventilator-day	7.9 ± 12.2	12.3 ± 15.4	5.5 ± 9.1	< 0.01
Mortality at distinct timepoints				< 0.01
In-hospital mortality	705 (10.4%)	705 (10.4%)	NA	NA
90-day mortality	1088 (16.1%)	1088 (16.1%)	NA	NA
1-year mortality	1648 (24.4%)	1648 (24.4%)	NA	NA

### Main pathogens among critically ill surgical patients

We found that 31.4% (2122/6748) of critically ill patients had at least one positive culture during the index admission, and the number of patients with positive culture in the blood, respiratory tract, urinary tract, skin and soft tissue and abdomen were 417, 1702, 554, 194 and 139, respectively (Table 2). The number of overall microbiological tests and proportions of a positive culture in distinct sites are listed in Additional file 1: Table S1. The majority of Gram-positive cocci was *Staphylococcus aureus* (*n* = 214, 44.4%), followed by *Enterococcus faecium* (*n* = 112, 23.2%) and *Enterococcus faecalis* (*n* = 76, 15.8%). Among the Gram-negative bacilli, the leading identified pathogens were *Pseudomonas aeruginosa* (*n* = 652, 35.7%), *Klebsiella pneumoniae* (*n* = 561, 30.7%), *Acinetobacter baumannii* (*n* = 347, 19.0%) as well as *Escherichia coli* (*n* = 276, 15.1%), and the data were consistent with the nationwide surveillance for main pathogens in healthcare facilities of Taiwan [20]. Fungal infection is increasingly a crucial issue among critically ill patients worldwide [21], and we found that 6.4% (435/6748) of critically ill surgical patients had positive fungal culture, with *Candida albicans* (*n* = 238, 54.7%) accounted for the majority of positive fungal culture, followed by *Candida glabrata* (*n* = 87, 20.0%) and *Candida tropicalis* (*n* = 46, 10.6%). In addition, 64 patients have a positive culture for *Aspergillus*, mainly in the respiratory tract.

**Table 2:** Pathogens identified in the cultures of 2122 patients during their index admission

	Total		Blood		Respiratory tract		Urinary tract		Skin and soft tissue		Abdomen	
	(*N* = 2122)		(*N* = 417)		(N = 1702)		(*N* = 554)		(*N* = 194)		(*N* = 139)	
	*n*	%	*n*	%	*n*	%	*n*	%	*n*	%	*n*	%
Gram-positive cocci	*N* = 482		*N* = 97		*N* = 195		*N* = 89		*N* = 49		*N* = 93	
*Staphylococcus aureus*	214	44.4%	36	37.1%	166	85.1%	3	3.4%	11	22.4%	5	5.4%
*Enterococcus faecium*	112	23.2%	20	20.6%	0	0.0%	20	22.5%	13	26.5%	20	21.5%
*Enterococcus faecalis*	76	15.8%	10	10.3%	0	0.0%	25	28.1%	11	22.4%	6	6.5%
Gram-negative bacilli	*N* = 1825		*N* = 283		*N* = 1530		*N* = 294		*N* = 136		*N* = 66	
*Pseudomonas aeruginosa*	652	35.7%	44	15.5%	538	35.2%	98	33.3%	31	22.8%	19	28.8%
*Klebsiella pneumoniae*	561	30.7%	68	24.0%	455	29.7%	47	16.0%	29	21.3%	16	24.2%
*Acinetobacter baumannii*	347	19.0%	46	16.3%	290	19.0%	10	3.4%	17	12.5%	5	7.6%
*Escherichia coli*	276	15.1%	45	15.9%	98	6.4%	106	36.1%	29	21.3%	16	24.2%
*Enterobacter cloacae*	186	10.2%	28	9.9%	123	8.0%	12	4.1%	13	9.6%	8	12.1%
*Serratia marcescens*	101	5.5%	15	5.3%	84	5.5%	2	0.7%	5	3.7%	0	0.0%
*Haemophilus influenzae*	70	3.8%	0	0.0%	70	4.6%	0	0.0%	0	0.0%	0	0.0%
*Enterobacter aerogenes*	65	3.6%	8	2.8%	52	3.4%	3	1.0%	4	2.9%	1	1.5%
Fungi	*N* = 435		*N* = 64		*N* = 122		*N* = 237		*N* = 24		*N* = 16	
*Candida albicans*	238	54.7%	23	35.9%	52	42.6%	129	54.4%	19	79.2%	11	68.8%
*Candida glabrata*	87	20.0%	10	15.6%	8	6.6%	55	23.2%	5	20.8%	4	25.0%
*Candida tropicalis*	46	10.6%	4	6.3%	5	4.1%	29	12.2%	2	8.3%	1	6.3%
*Candida parapsilosis*	7	1.6%	4	6.3%	0	0.0%	2	0.8%	0	0.0%	0	0.0%
*Candida krusei*	3	0.7%	0	0.0%	0	0.0%	1	0.4%	0	0.0%	0	0.0%
Yeast	42	9.7%	4	6.3%	1	0.8%	129	54.4%	0	0.0%	0	0.0%
*Aspergillus*	64	14.7%	22	34.4%	50	41.0%	2	0.8%	0	0.0%	0	0.0%

### Long-term mortality impact of positive culture in critically ill surgical patients

To further assess the association of positive clinical cultures with long term, we included positive culture from any sites as one of the predictors in a multivariable Cox proportional hazards model (Table 3). We found that a positive culture from any sites was independently associated with a 57.9% increased hazard of long-term mortality, after adjusting for age (aHR, 1.019; 95% CI 1.016–1.022), male gender (aHR, 1.175; 95% CI 1.063–1.281), diabetes mellitus (aHR, 1.171; 95% CI 1.062–1.291), congestive heart failure (aHR, 1.370; 95% CI 1.172–1.601), moderate or severe liver disease (aHR, 1.326; 95% CI 1.077–1.633), end-stage renal disease (aHR, 1.434; 95% CI 1.007–2.042), presence of malignancy (aHR, 2.155; 95% CI 1.929–2.408), and presence of metastatic tumour (aHR, 4.553; 95% CI 3.867–5.360). With regard to the impact of surgical types, we found higher long-term mortality in patients receiving major abdomen surgery (aHR, 1.410; 95% CI 1.207–1.647) and neurosurgery (aHR, 1.527; 95% CI 1.276–1.828) compared with those receiving cardiovascular surgery. Moreover, higher APACHE II score (aHR, 1.064; 95% CI 1.056–1.073), presence of shock (aHR, 1.401; 95% CI 1.273–1.542), presence of early fluid overload (aHR, 1.031; 95% CI 1.016–1.047, per 1 L increment) and receiving renal replacement therapy (aHR, 1.966; 95% CI 1.693–2.284) were associated with an increased risk of long-term overall mortality, whereas a higher BMI appeared to be a protective factor (aHR, 0.955; 95% CI 0.946–0.965). In subgroup analyses, the association between culture positivity and mortality appeared to be stronger in female patients, patients without malignancy or metastatic tumour, and patients who received cardiovascular surgery (Additional file 1: Table S2). Moreover, we found that culture positivity, defined by distinct numbers of pathogens and cites, was associated with increased long-term mortality at a dose–response manner (Table 4).

**Table 3:** Cox proportional hazards regression for mortality

Characteristics	Univariable		Multivariable	
	HR (95% CI)	*p* value	HR (95% CI)	*p* value
Basic characteristics				
Age, per 1 year increment	1.029 (1.026–1.032)	< 0.001	1.019 (1.016–1.022)	< 0.001
Male gender	1.296 (1.184–1.419)	< 0.001	1.167 (1.063–1.281)	0.001
Body mass index, pre 1 increment	0.947 (0.937–0.956)	< 0.001	0.955 (0.946–0.965)	< 0.001
Comorbidities				
Diabetes mellitus	1.292 (1.176–1.419)	< 0.001	1.171 (1.062–1.291)	0.001
Congestive heart failure	1.272 (1.100–1.470)	0.001	1.370 (1.172–1.601)	< 0.001
Chronic pulmonary disease	1.819 (1.482–2.232)	< 0.001	1.166 (0.942–1.442)	0.158
Moderate or severe liver disease	2.002 (1.635–2.452)	< 0.001	1.326 (1.077–1.633)	0.008
End-stage renal disease	1.841 (1.299–2.610)	< 0.001	1.434 (1.007–2.042)	0.046
Malignancy	2.435 (2.227–2.663)	< 0.001	2.155 (1.929–2.408)	< 0.001
Presence of metastatic tumour	3.728 (3.198–4.344)	< 0.001	4.553 (3.867–5.360)	< 0.001
Types of surgery				
Cardiovascular surgery	Reference		Reference	
Neurosurgery	1.454 (1.266–1.670)	< 0.001	1.410 (1.207–1.647)	< 0.001
Major abdominal surgery	4.013 (3.448–4.671)	< 0.001	1.527 (1.276–1.828)	< 0.001
Severity and managements				
APACHE II, per 1 increment	1.115 (1.107–1.123)	< 0.001	1.064 (1.056–1.073)	< 0.001
Presence of shock	1.994 (1.831–2.172)	< 0.001	1.401 (1.273–1.542)	< 0.001
Receiving mechanical ventilation	2.300 (2.115–2.501)	< 0.001	1.079 (0.969–1.201)	0.166
Fluid overload, days 1–3, per 1-L increment	1.104 (1.087–1.120)	< 0.001	1.031 (1.016–1.047)	< 0.001
Receiving renal replacement therapy	4.372 (3.831–4.989)	< 0.001	1.966 (1.693–2.284)	< 0.001
Positive culture during admission	2.842 (2.614–3.091)	< 0.001	1.579 (1.422–1.754)	< 0.001

**Table 4:** Sensitivity analysis in the estimation of the mortality risk for distinct definitions of culture positivity

Distinct definitions of culture positivity	aHR* (95%CI)
Pathogens	
At least one pathogen (main finding)	1.56 (1.38–1.76)
At least two pathogens	1.94 (1.66–2.26)
At least three pathogens	1.97 (1.71–2.27)
Culture sites	
At least one site (main finding)	1.56 (1.39–1.75)
At least two sites	1.99 (1.72–2.30)
At least three sites	2.38 (2.00–2.82)

*Adjusted covariates including variables listed in Table 3. *aHR* adjusted hazard ratio, *CI* confidence interval

## Discussion

Identification of early determinants for long-term mortality in critically ill surgical patients is currently a research niche. We linked the critical care database at TCVGH with the nationwide death registration data in Taiwan to investigate the association between culture positivity and long-term mortality in critically ill surgical patients. We found that positive culture from any sites was independently associated with the long-term mortality after adjusting relevant covariates. These findings provide evidence for the prolonged impact of culture positivity during admission and indicate the need for vigilance among critically ill surgical patients with a positive culture during admission.

Due to the steady improvement of acute mortality and increased awareness of the prolonged impact of critical illness on surgical patients, the long-term outcome is currently an essential issue in critically ill surgical patients [2, 3, 22]. Wunsch et al., analysing 3 year outcome among 35,308 patients who were discharged from ICU and two control groups, including 12,173 hospital controls 5266 general controls in Medicare claim, reported that patients discharged from ICU had a consistent higher 3-year mortality compared with those in hospital controls (39.5% vs. 34.5%; aHR 1.07, 95% CI 1.01–1.10) and general controls (39.5% vs. 14.9%; aHR 2.39, 95% CI 2.31–2.48) after adjustment of relevant covariates [4]. de Lima et al*.* conducting a multicenter study in Brazil with 108,302 patients requiring ICU admission and 216,292 non-ICU admission controls, found that patients admitted to the ICU were more likely to be readmitted to hospital (25.4 vs. 17.4%, *p* < 0.001) and to ICU (31.4 vs. 7.3%, *p* < 0.001) compared with the non-ICU admission control group [6]. de Lima et al*.* found not only that the post-ICU 1-year mortality after discharge tended to be higher in ICU patients than those in the non-ICU control group (14.3 vs. 3.9%, *p* < 0.001), but also a significant interaction between surgical status and mortality, with lower mortality in surgical patients (aHR 2.7, 95% CI 2.5–2.9) than those in medical patients (aHR 3.4, 95% CI 3.3–3.5) [6]. Similar to our data, Brakenridge et al*.* recently addressed the long-term outcome in 301 critically ill surgical patients with sepsis at one trauma centre of the University of Florida during 2016–2018 and reported the 30-day mortality and 1-year mortality was 9.6 and 20.9%, respectively [3]. The aforementioned data with regard to the long-term outcome in critically ill surgical patients highlight an essential need to explore the long-term outcome and to identify early determinants for long-term mortality in critically ill surgical patients.

Currently, there are discordant data with regard to the association between culture positivity and outcome in critically ill patients. Notably, few studies addressed the long-term impact of culture positivity, and the research focused on critically ill surgical patients is particularly sparse. Kethireddy et al*.* conducting a multicentre retrospective nested cohort study during 1997–2010 in 8670 patients with septic shock, reported that 30.6% of patients had a positive culture and culture positivity appeared unrelated with the hospital mortality (52.7 vs. 52.9%, *p* = 0.976) [12]. Similarly, Li et al. recently performed a meta-analysis including 22,655 patients of 7 studies and reported that culture-positive appeared to be unrelated with short-term mortality (OR: 0.95; 95% CI 0.88–1.01), although the heterogeneity among studies was apparently high (*I*^2^ = 80%) [14]. Recently, Liu et al*.* using the inverse probability of treatment weighting method to investigate the impact of positive blood culture on 30 and 90-day mortality among 1405 patients with suspected sepsis, found that blood culture-positive patients had similar 30-day mortality but higher 90-day mortality than those in culture-negative patients [16]. The aforementioned data showed that culture positivity appears to be associated with long-term outcomes instead of short-term outcomes; therefore, there is an essential need for studies focusing on long-term outcomes in critically ill patients. Our previous study found a long-term mortality impact of culture positivity in 638 critically ill cancer patients receiving surgery during ICU admission [15]. In the present study, we enrolled a relatively large number of patients who were admitted to surgical ICUs and provide evidence that culture positivity affects the long-term mortality in critically ill patients. Collectively, these evidence point out the previously ignored role of culture positivity in the long-term outcome among critically ill surgical patients.

A number of studies have explored the association between the site of infection and outcome; however, the majority of studies focused on short-term outcome [23, 24]. Moreover, the data in critically surgical patients remain sparse [11, 25]. In addition to the association between long-term mortality and the positive culture of any sites, we also identified that bloodstream infection, positive culture in the respiratory tract and positive culture in the urinary tract were independent determinants for long-term mortality (Additional file 1: Fig. S1 and Table S3). The trends of association between long-term mortality and positive culture in the abdomen as well as skin/soft tissue were similar with those in other culture cites, although not reach statistical significance mainly due to the relatively small case number of patients with positive culture in abdomen/skin and soft tissue. In line with our finding that patients with bloodstream infection appear to have poor long-term survival, Sortz et al. recently investigated the association between infection sites and long-term mortality in 316 critically ill surgically patients, with the majority (140/316, 44.3%) of enrolled subjects were those with abdominal infection, at the Shands Hospital in the United States and found that those with bloodstream infection including vascular infection tended to have higher 1-year mortality than those with abdominal and pulmonary infection, with the 1-year mortality of bloodstream infection, abdominal infection and pulmonary infection were 43, 26, and 20%, respectively [25]. Notably, Cox et al. recently explored the prolonged immunological and metabolic alternation among 144 critically ill surgical patients with abdominal sepsis at the trauma centre of Florida University [11]. In brief, Cox et al. found that 37% of patients with surgical sepsis developed chronic critical illness, defined by persistent organ dysfunction and the requirement of ICU care for longer than 14 days [11, 22]. Those with CCI had a higher level of day-14 biomarkers, including inflammatory biomarkers (Interleukin (IL)-6, IL-8), metabolic abnormality relevant biomarker (glucagon-like peptide 1 (GLP-1) as well as cell death-associated biomarker (soluble programmed death ligand one (sPDL1)), and the 1-year mortality was higher in those with CCI than that in non-CCI controls (42 vs. 7%, *p* < 0.05) [11]. These evidence indicate the previously ignored prolonged impact of infection during admission on the long-term outcome in critically ill surgical patients.

The association between culture positivity and long-term outcome in critically ill surgical patients might result from the critical illness-associated pathophysiological alterations, including the alternation of microbiota [26, 27]. Morgan A. recently summarised that the increased long-term mortality among ICU survivors may be attributed to multisystem pathophysiological alterations, including airway impairment result from prolonged intubation, ICU acquired weakness, delirium, and altered gastrointestinal function including dysphagia, anorexia, diarrhoea as well as altered microbiota [26]. As we have identified in the present study, the early predictors for long-term mortality should provide evidence for risk stratification and early intervention, such as the implementation of physical, psychological and nutritional support, among critically ill surgical patients during and after the ICU admission [28]. Furthermore, the culture positivity may potentially reflect the alternation of microbiota, and accumulating evidence have shown the crucial role of microbiota among critically ill patients in the past decade [8, 27]. Freedberg et al*.* prospectively performed rectal surveillance swabs using 16S rRNA gene sequencing at ICU admission in 301 critically ill patients found that early disruption of microbiota by Enterococcus domination was correlated with an increased risk for 30-day death or infection by 22% [29]. In addition, increasing evidence have found that the alternation of microbiota, so-called dysbiosis, may have an impact on the outcome of critically ill patients for at least 3 months [7, 9, 30]. Darden et al. used single-cell RNAseq and myeloid-enriched peripheral blood mononuclear cells among critically ill surgical patients on day-21 after the sepsis to characterise the immunosuppressive transcriptome of myeloid-derived suppressor cells in critically ill surgical patients with CCI [31, 32]. We speculate that alternation of microbiota might account for the relatively strong association between culture positivity and risk of mortality among non-infectious critically ill surgical patients, including those receiving cardiovascular surgery and patients without malignancy or metastatic tumour (Additional file 1: Table S2). Notably, in addition to the straightforward effect of antibiotics on the constitution of microbiota, a number of medications, including proton pump inhibitors, nonsteroidal anti-inflammatory drugs and opioids, have been found to essential impact on gut microbiota among critically ill patients [10]. Intriguingly, recent studies in patients receiving cardiovascular surgery and patients with coronavirus disease 2019 (COVID-19) infection reported that the microbiome may be gradually restored approximately 6 months after critical illness [9, 33], and the finding appears to be in line with our data that the mortality impact of culture positivity mainly existed within 6–9 months. Collectively, these evidence point out the prolonged impact of infection and dysbiosis during ICU admission on the long-term outcome in critically ill surgical patients.

There are limitations in the present study. First, a single-centre study and external validation is required. However, we enrolled a relatively high number of subjects, and the data used in the present study are data obtained in routine care in surgical ICUs. Therefore, we think the issue regarding generalisation should largely be mitigated. Second, we could not ascertain cause-of-death and functional status, because the used claim database did not contain these information. Third, due to the observational nature of this study, we were unable to make causal inferences with regard to how microbial factors attribute to long-term mortality. Fourth, the decision for microbiological surveillance and indication of ICU admission were made by the individual attending physician that might introduce a confounding effect. However, the administration of intensivists in the study ICUs should at least partly mitigate the aforementioned concern. Moreover, some potential confounders, such as duration of central line and concomitant medications including corticosteroid/immunosuppressants, were unavailable given that we have adjusted the majority of variables in critical care.

## Conclusion

The long-term outcome is an emerging research priority in critically ill surgical patients. In the present study, we linked two databases to explore the association between culture positivity and long-term mortality in critically ill surgical patients. We identified that a positive culture from any sites was independently correlated with a 54.5% increased hazard of long-term mortality after adjustment of relevant covariates. Our findings highlight the crucial role of culture positivity in critically ill patients, and more mechanistic studies are warranted to elucidate the underlying mechanisms.

## Supplementary Information

**Additional file 1: Figure S1. **Adjusted hazard ratios for mortality categorised by culture sites. **Table S1.** Number of microbiological tests and proportion of positive culture in distinct culture sites. **Table S2. **Effect modification of variables on the association between culture positivity and risk of mortality. **Table S3. **Cox proportional hazards regression for mortality categorised by culture sites.

## Data Availability

The data underlying this article will be shared on reasonable request to the corresponding author.
