# Supplementary material for: The association between culture positivity and long-term mortality in critically ill surgical patients
Source: J Intensive Care. 2021 Oct 26;9:66. doi: 10.1186/s40560-021-00576-2 (PMC8546784; doi:10.1186/s40560-021-00576-2)
Supplement: Supplementary file 1 — Additional file 1: Figure S1. Adjusted hazard ratios for mortality categorised by culture sites. Table S1. Number of microbiological tests and proportion of positive culture in distinct culture sites. Table S2. Effect modification of variables on the association between culture positivity and risk of mortality. Table S3. Cox proportional hazards regression for mortality categorised by culture sites. [file 40560_2021_576_MOESM1_ESM.pdf]

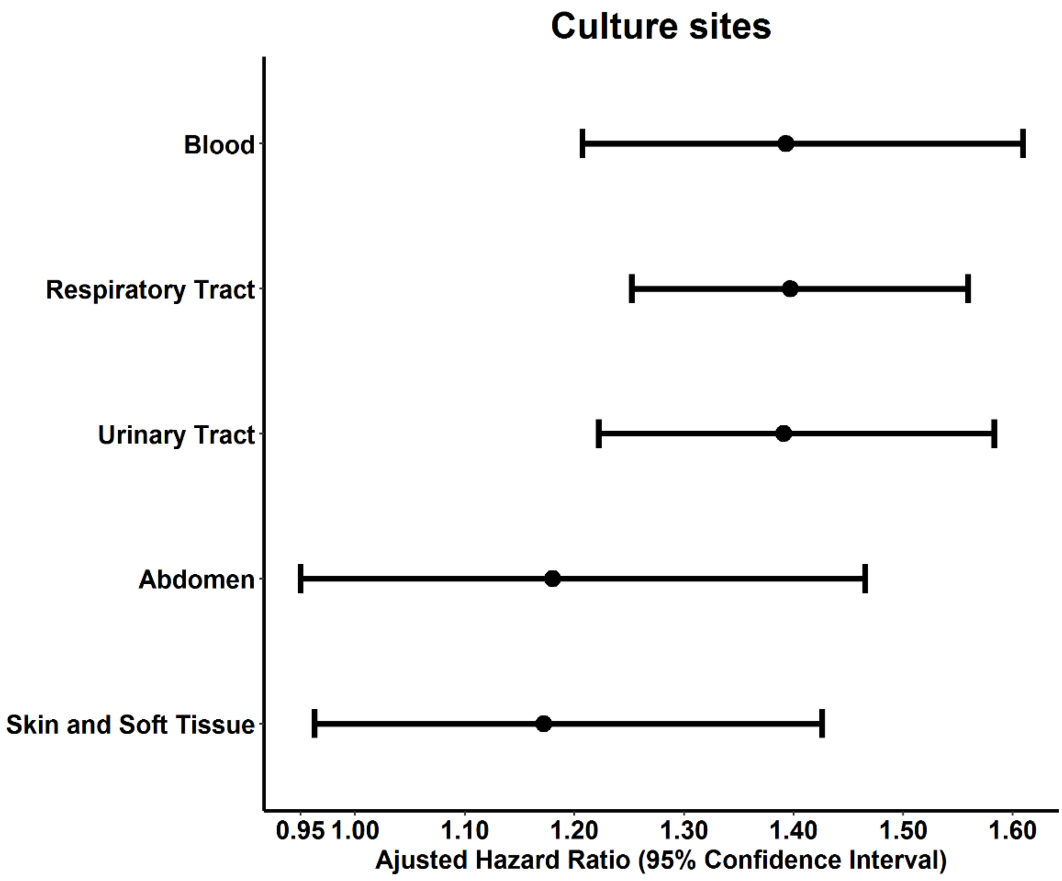

Supplemental Figure 1. Adjusted hazard ratios for mortality categorised by culture sites

Supplemental table 1. number of microbiological tests and proportion of positive culture in distinct culture sites.

	Blood	Respiratory tract	Urinary tract	Skin and soft tissue	Abdomen
**All tests**	3,069	3,629	2,445	243	423
**Positive tests**	417	1,702	554	194	139
**Positive (%)**	13.6%	46.9%	22.7%	79.8%	32.9%

Supplemental table 2. Effect modification of variables on the association between culture positivity and risk of mortality

Variables	Crude HR (95%CI)	p-value	Adjusted HR (95% CI)	p-value
**Age group**		0.897		0.063
≤ 40 years	2.515 (1.736-3.643)		1.384 (0.860-2.227)	
40-64 years	2.851 (2.494-3.259)		1.587 (1.347-1.868)	
≥ 65 years	2.791 (2.491-3.126)		1.637 (1.419-1.889)	
**Gender**		0.005		0.014
Female	3.360 (2.888-3.909)		1.762 (1.451-2.140)	
Male	2.618 (2.367-2.897)		1.563 (1.378-1.773)	
**Diabetes**		0.270		0.156
No	2.896 (2.624-3.196)		1.682 (1.486-1.904)	
Yes	2.619 (2.231-3.076)		1.507 (1.232-1.843)	
**Malignancy**		<0.001		<0.001
No	3.108 (2.806-3.444)		1.708 (1.499-1.946)	
Yes	2.185 (1.886-2.532)		1.590 (1.334-1.896)	
**Presence of metastatic tumour**		0.003		0.003
No	2.930 (2.684-3.199)		1.679 (1.505-1.873)	
Yes	2.078 (1.543-2.799)		1.151 (0.7531-1.76)	
**Types of surgery**		0.001		<0.001
Cardiovascular surgery	4.488 (3.499-5.758)		2.087 (1.490-2.924)	
Neurosurgery	2.399 (2.093-2.750)		1.386 (1.156-1.662)	
Major abdomen surgery	1.951 (1.610-2.365)		1.689 (1.355-2.105)	

HR: hazard ratio; C.I.: confidence interval

Supplemental table 3. Cox proportional hazards regression for mortality categorised by culture sites.

Characteristics	Univariable		Multivariable	
	**HR (95% C.I.)**	***p* value**	**HR (95% C.I.)**	***p* value**
Age, per 1 year increment	1.029 (1.026-1.032)	<0.001	1.012 (1.009-1.015)	<0.001
Male gender	1.296 (1.184-1.419)	<0.001	1.243 (1.133-1.364)	<0.001
Body mass index, pre 1 increment	0.947 (0.937-0.956)	<0.001	0.940 (0.930-0.949)	<0.001
Charlson comorbidity index, per 1 increment	1.469 (1.433-1.505)	<0.001	1.309 (1.272-1.346)	<0.001
APACHE II, higher than 25	1.115 (1.107-1.123)	<0.001	1.857 (1.681-2.051)	<0.001
Presence of shock	1.994 (1.831-2.172)	<0.001	1.425 (1.303-1.559)	<0.001
Receiving mechanical ventilation	2.300 (2.115-2.501)	<0.001	1.109 (0.995-1.235)	0.061
Fluid overload, day 1-3, per 1L increment	1.104 (1.087-1.120)	<0.001	1.036 (1.020-1.052)	<0.001
Receiving renal replacement therapy	4.372 (3.831-4.989)	<0.001	1.626 (1.401-1.888)	<0.001
End-stage renal disease	1.841 (1.299-2.610)	0.001	1.057 (0.742-1.505)	0.758
Positive culture				
Blood	3.294 (2.906-3.735)	<0.001	1.445 (1.251-1.669)	<0.001
Respiratory tract	2.572 (2.362-2.801)	<0.001	1.382 (1.239-1.541)	<0.001
Urinary tract	2.768 (2.465-3.108)	<0.001	1.445 (1.269-1.646)	<0.001
Skin and soft tissue	2.635 (2.196-3.162)	<0.001	1.164 (0.957-1.416)	0.127
Abdomen	3.204 (2.607-3.938)	<0.001	1.128 (0.910-1.398)	0.272

HR: hazard ratio; C.I.: confidence interval; APACHE: acute physiology and chronic health evaluation.
